# Complete remission after single agent blinatumomab in a patient with pre-B acute lymphoid leukemia relapsed and refractory to three prior regimens: hyperCVAD, high dose cytarabine mitoxantrone and CLAG

**DOI:** 10.1186/s40164-016-0051-4

**Published:** 2016-07-26

**Authors:** Katherine Linder, Deepthi Gandhiraj, Madhura Hanmantgad, Karen Seiter, Delong Liu

**Affiliations:** Department of Medicine, New York Medical College and Westchester Medical Center, Valhalla, NY 10595 USA

## Abstract

**Background:**

The current standard of care for relapsed and refractory acute lymphoblastic leukemia (ALL) is combination chemotherapy.

**Case presentation:**

We report a case of highly refractory ALL who was treated with blinatumomab. The ALL in this patient relapsed within a month after completion of hyperCVAD regimen and was refractory to high dose mitoxantrone/cytarabine and CLAG regimens.

**Conclusion:**

This highly refractory pre-B Ph^−^ ALL was induced to complete remission after one course of single agent blinatumomab.

## Background

Adults with relapsed or refractory precursor B acute lymphoblastic leukemia have an unfavourable prognosis. The current standard of care for relapsed or refractory acute lymphoblastic leukemia (ALL) is combination chemotherapy which yields complete remission (CR) in 30–45 % of patients [1–5]. Cancer immunotherapy is being widely used nowadays for solid tumors as well as lymphomas. Chimeric antigen receptor-engineered T cells showed promise in the treatment of aggressive ALL and chronic lymphoid leukemia [6–9]. Blinatumomab is a bispecific T cell engager (BiTE) diabody construct with dual specificity for CD19 and CD3 [10–13]. Blinatumomab simultaneously binds CD3-positive cytotoxic T cells and CD19-positive B cells, resulting in T-cell-mediated serial lysis of normal and malignant B cells [13]. Thus, blinatumomab represents an immunotherapy that engages patients’ endogenous T cells to attack and potentially eradicate B-precursor ALL blasts. Blinatumomab was first reported in a clinical phase I trial in 38 patients with refractory non-Hodgkin lymphoma [11]. Blinatumomab has demonstrated promising activity and a favorable safety profile in relapsed/refractory (R/R) ALL and in ALL with minimal residual disease (MRD). A large, multicenter, phase II trial (MT103-211, NCT01466179) assessed blinatumomab in 189 adult patients with relapsed or refractory B cell ALL with Philadelphia chromosome (Ph) negativity [14]. 43 % of patients achieved CR or CRh within two cycles of treatment with the single-agent blinatumomab. Median relapse-free survival was 5 to 9 months for those patients who achieved CR/CRh [14]. However, in this trial, only 17 % of patients had more than two prior regimens.

We report here a highly refractory case of relapsed Ph^−^ pre B ALL who achieved CR with a single cycle of blinatumomab.

### Case report

The patient is a 32 year old female at 24 weeks of gestation who presented in January 2014 with bilateral submandibular swelling and pain with radiation to head, neck, chest, and back for 2 weeks. On her initial presentation she was found to have WBC 164 × 10^9^/L with a differential of 94.4 % lymphoblasts, platelets 27 × 10^9^/L, and hemoglobin of 10.4 gm/dL. A peripheral blood flow cytometry revealed abnormal immature B cell population that comprised 90 % of the total cells and were positive for CD10, CD19, CD22, CD34, Tdt, but negative for CD 20. These findings were characteristic for precursor B-cell ALL. Cytogenetics was negative for Philadelphia chromosome. Karyotyping showed a normal 46XX chromosome display. FISH was negative for BCR/ABL and MLL gene rearrangement on chromosome 11q23.

Due to the pregnancy status, she underwent chemotherapy induction with the modified Linker regimen in 01/2014 [15–17]. This included cyclophosphamide (1 gm/m^2)^ on day 1, vincristine 2 mg IV on days 1, 8, 15, 22, 30, 37, and 44; daunorubicin 50 mg/m^2^ on days 1–3, 30 and 31, and prednisone 100 mg po days 1–4, 80 mg on days 5–28, tapered slowly to off on day 62. Cerebral spinal fluid was negative for malignant cells.

On day 38, she had a bone marrow biopsy revealing a hypocellular marrow, erythroid hyperplasia, dyserythropoiesis and atypical immature B-cells consistent with residual disease of precursor B-ALL. Concurrent flow cytometry of the aspirate showed an immature B-cell population (1.8 % of total B cells) expressing CD19, Tdt. Clinically she was in complete remission by standard definition with platelets 140 × 10^9^/L absolute neutrophil count (ANC) 1.43 × 10^9^/L.

She had C-section at gestation week 34 and delivered a healthy girl.

The second cycle of chemotherapy began 1 week post-partum with standard hyperCVAD regimen B in 3/2014 [18, 19]. She had Ommaya reservoir placed and received intrathecal methotrexate twice per cycle following the standard protocol defined in the hyperCVAD regimen. This was followed by six more cycles of hyperCVAD. Shortly after completion of last hyperCVAD, she started to have progressively worsening cytopenia. In November 2014, a bone marrow aspiration and biopsy were done to evaluate her disease status. The flow cytometry study of the aspirate showed a 79 % lymphoblast population expressing the following markers: CD19, CD10, Tdt, and CD34. This was consistent with full relapse of her precursor B-cell ALL. Karyotyping was 46XX. The patient received re-induction chemotherapy with high dose cytarabine (3 gm/m^2^ daily IV over 3 h × 5), and mitoxantrone (60 mg/m^2^ once) [20].

A repeat bone marrow aspirate and biopsy was performed in 1/2015 and revealed 43 % lymphoblasts. This confirmed persistent refractory precursor-B ALL. Salvage chemotherapy with CLAG regimen (cladribine, cytarabine and G-CSF) was started immediately [21–23]. Four weeks later, a repeat bone marrow biopsy showed 68 % lymphoblasts, consistent with refractory disease to the CLAG regimen.

In February 2015, she was treated with blinatumomab. She was started with 9 mcg/day. On day 3 of blinatumomab therapy, the patient started spiking fever to 102.7 °F, with tachycardia and hypotension. The patient was transferred to the ICU for closer monitoring. Chemotherapy with blinatumomab was temporarily held for suspected cytokine release syndrome (CRS). The patient received one dose of tocilizumab 8 mg/kg IV once in addition to methylprednisolone. She required aggressive fluid resuscitation and was on vasopressors for 2 days. Blinatumomab was restarted 2 days later at 9 mcg/day. In total she received 9 days of blincyto at 9 mcg daily. The dose was increased to 28 mcg per day and continued to day 28. Her blood counts improved steadily. A bone marrow biopsy was performed. Flow cytometry of the aspirate revealed no immature B-cell population. Pathology revealed trilineage hematopoiesis with no increase in blasts. A concurrent peripheral blood counts showed platelets of 247 × 10^9^/L and ANC > 1 × 10^9^/L. At this point, the patient had a pathology documented complete remission (CR) after one course of single agent blinatumomab.

Since she was not eligible for allogeneic hematopoietic stem cell transplantation (HSCT), maintenance chemotherapy with MTX, vincristine, pegylated asparaginase and dexamethasone was given. However, she developed acute pancreatitis after four cycles of the PEG-asparaginase-containing regimen. Upon recovery from the pancreatitis, a bone marrow biopsy confirmed that the patient remained in CR in August 2015.

In September 2015, she was started on a 2-week cycle of blinatumomab as maintenance. Due to lack of convincing scientific data for using blinatumomab as maintenance treatment, further treatment with blinatumomab was stopped. She maintained normal blood counts and had normal activity. Unfortunately, in February 2016, her WBC increased to 16.4 × 10^9^/L, platelet counts decreased to 75 × 10^9^/L. Bone marrow biopsy at this point showed an abnormal immature B-cell population expressing CD19 (dim), CD22, CD10, CD34, TdT, HLA-DR, CD9 and CD38 (heterogeneous). The findings were consistent with relapsed preB-ALL. Since CD19 was still positive, blinatumomab was started. Two weeks later, the WBC rose to 74.4 × 10^9^/L, platelets down to 19 × 10^9^/L. Peripheral blood had 71 % blasts. Therefore, her ALL was refractory to blinatumomab re-treatment. At the time of this report, she was receiving additional treatment.

## Discussion

The ALL in this patient relapsed quickly after completion of the hyperCVAD regimen and was refractory to high dose mitoxantrone/cytarabine and CLAG regimens. This highly refractory pre-B Ph^−^ ALL was induced to complete remission after one course of single agent blinatumomab. This is significant since there has been no case like this in our historical database that achieved CR. The median survival in this patient population is traditionally around 3 months.

Blinatumomab represents the first-in-class BiTE antibody approved by FDA for clinical use. This novel diabody opens a new channel of immunotherapy for patients with relapsed/refractory B cell ALL. However, the requirement for continuous IV infusion due to the small molecular weight and rapid clearance presents a challenge for clinical application. Newer tetravalent bispecific antibodies, AFM11 and AFM13, are being tested in clinical trials as weekly or twice weekly infusion [24, 25]. T cells with CD19/CD3 chimeric antigen receptors (CAR-T19) have been shown to induce high remission rate (90 % CR in refractory ALL) [9, 26]. The rate of severe CRS associated with CAR-T therapy was reported to be 27 %. This rate was much higher than that of blinatumomab (2 %) [14]. CAR-T penetrates blood–brain barrier [9]. It remains unknown whether blinatumomab has similar property. The optimal treatment duration and schedule of blinatumomab for patients who can not receive allogeneic HSCT remain to be determined. The role of consolidation or maintenance therapy with blinatumomab also remains an area of investigation [13].

The patient had a prolonged remission after blinatumomab and PEG-asparaginase-containing regimen, but eventually relapsed and became refractory to blinatumomab re-treatment. One report found that PD-L1 overexpression developed in the ALL cells refractory to blinatumomab [27]. Nivolumab was known to be highly active in refractory Hodgkin lymphoma [28]. It is unclear yet whether ALL relapsed after blinatumomab might respond to PD-1 antibodies. Novel agents with different mechanisms of action, such as ABT-199 (venetoclax) and ACP-196 (acalabrutinib) are being studied for highly refractory lymphoid malignancies [13, 29]. New therapeutic options are urgently needed for highly refractory ALL patients.

## Abbreviations

CRS: cytokine release syndrome
CLAG: cladribine, AraC, G-CSF
CVAD: cyclophosphamide, vincristine, Adriamycin, dexamethasone
HSCT: hematopoietic stem cell transplantation
MTX: methotrexate
